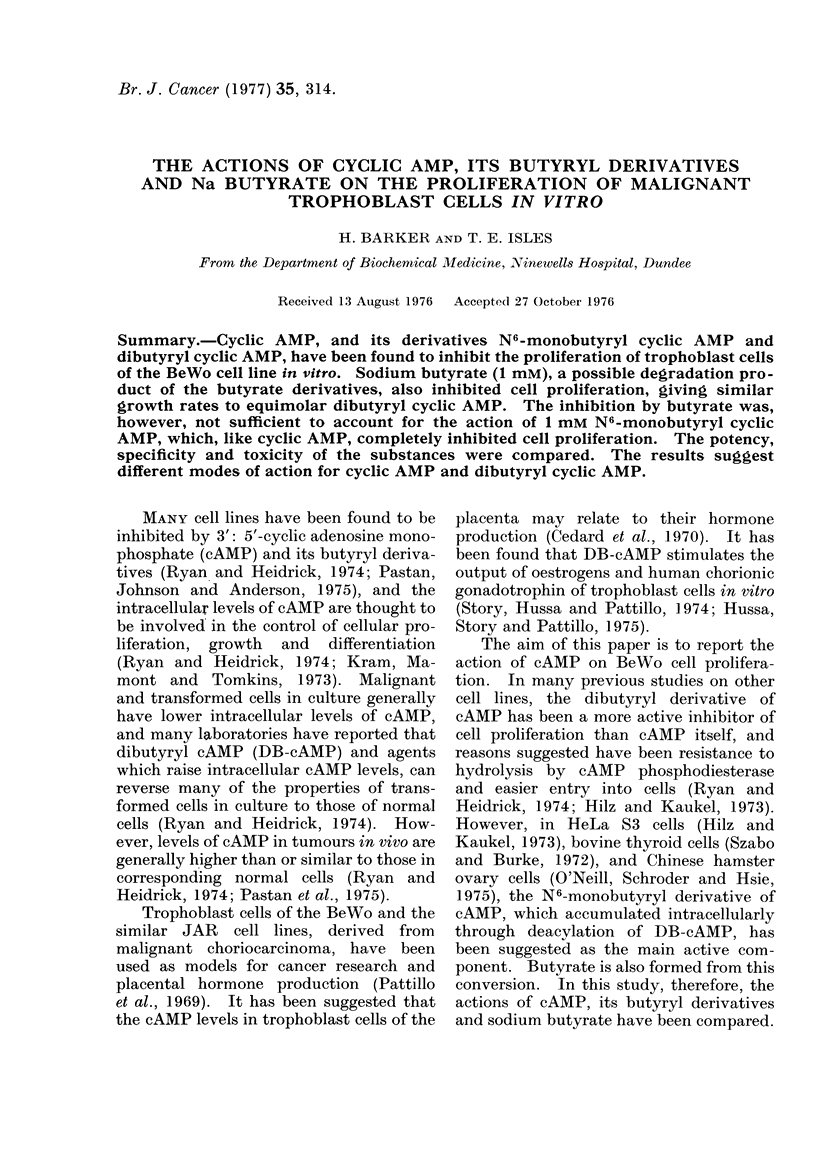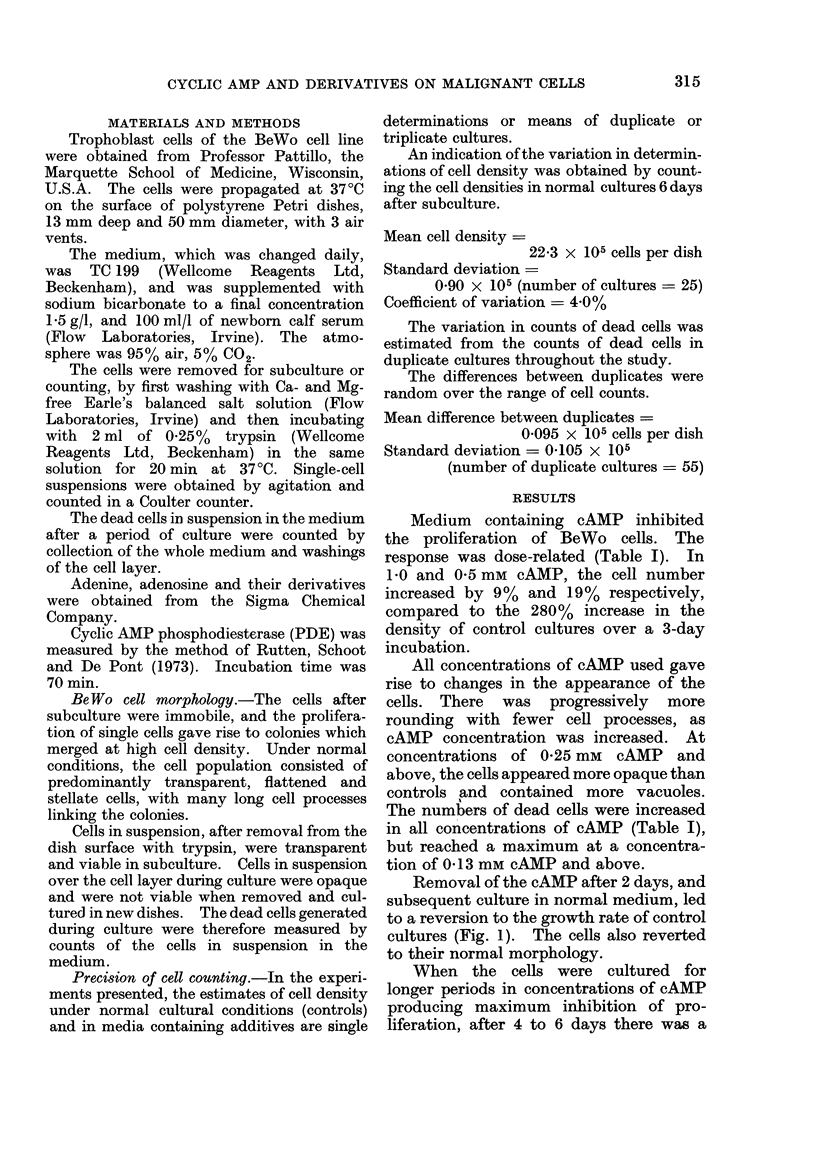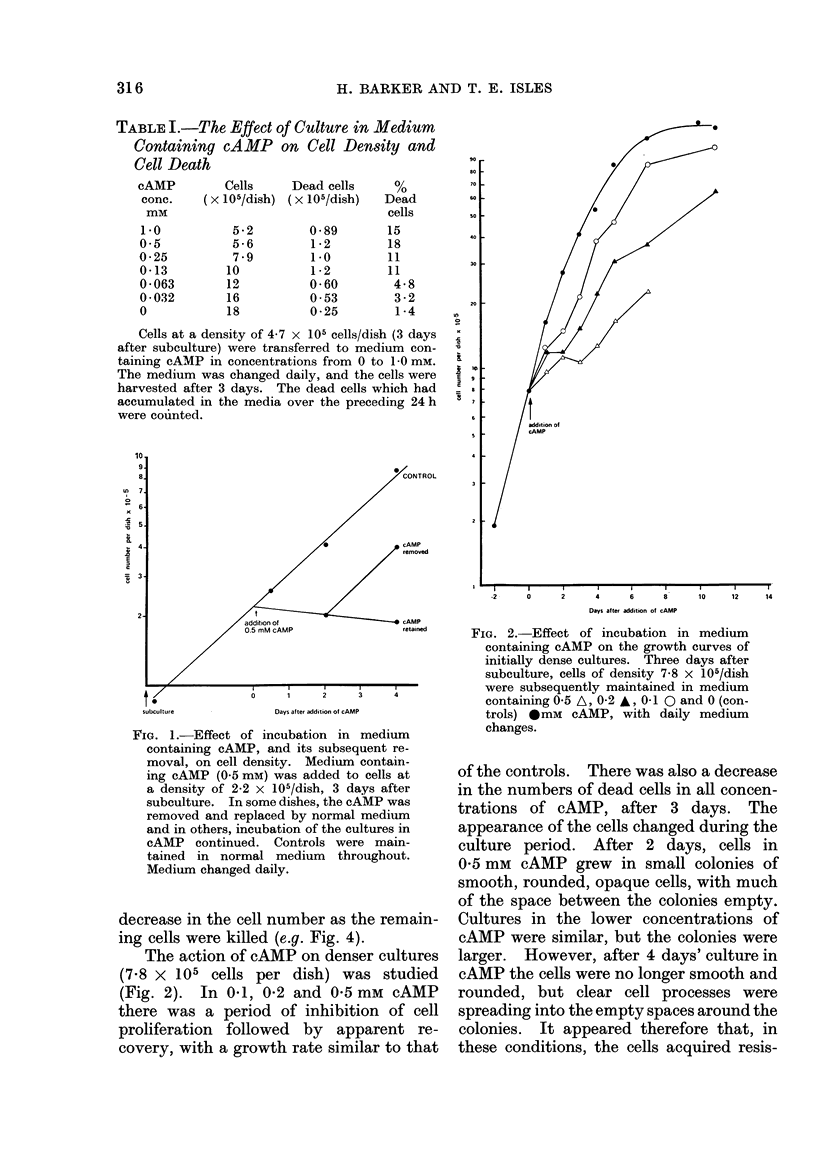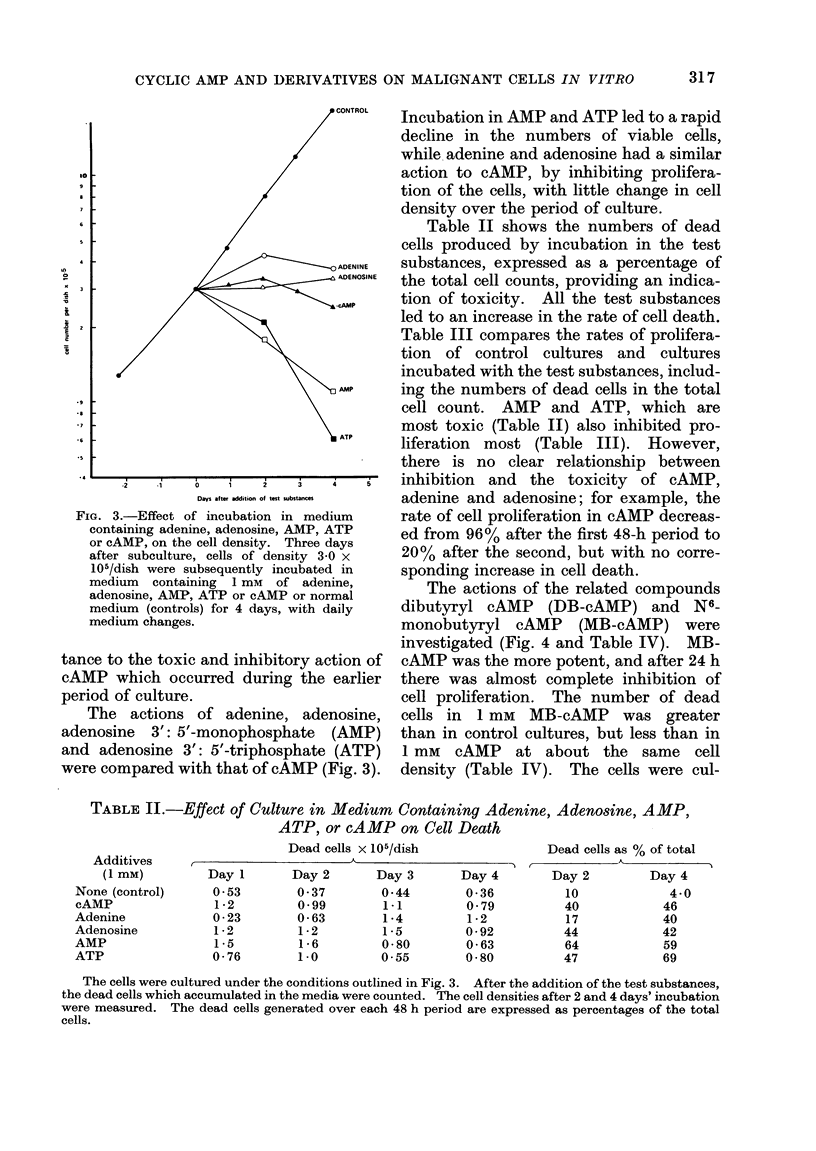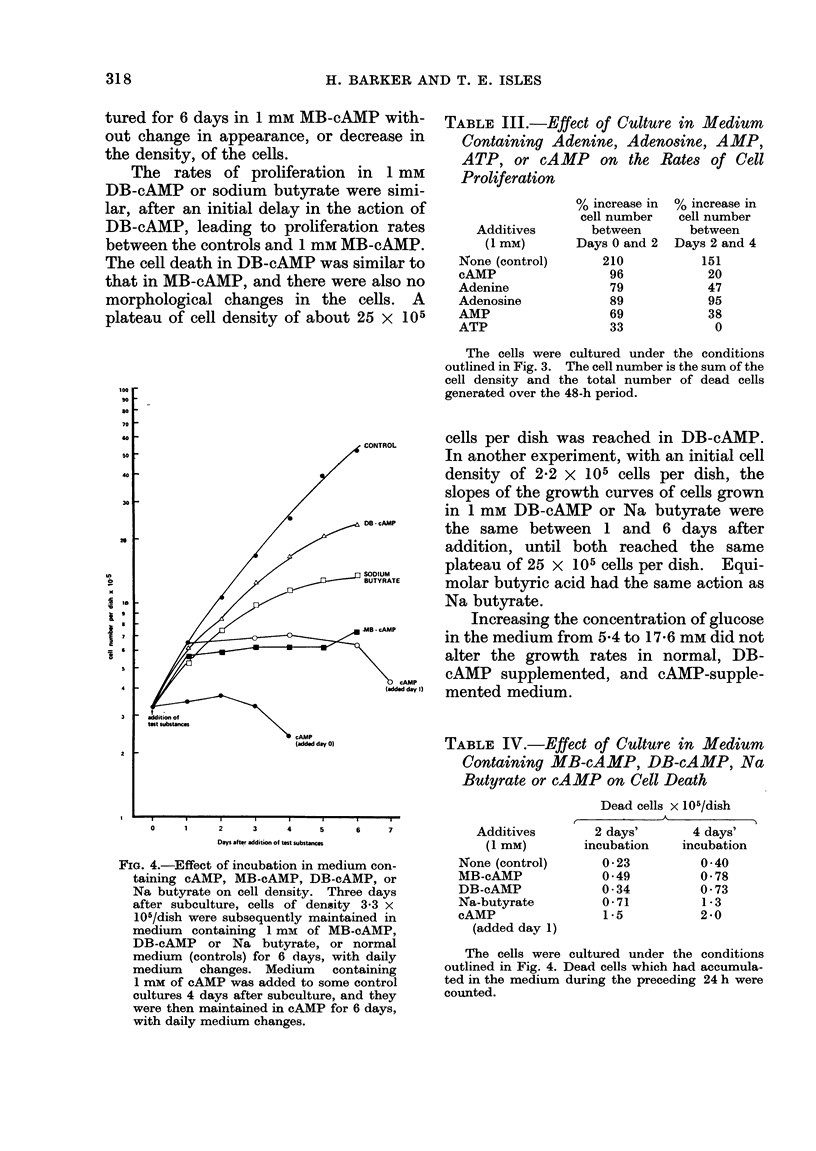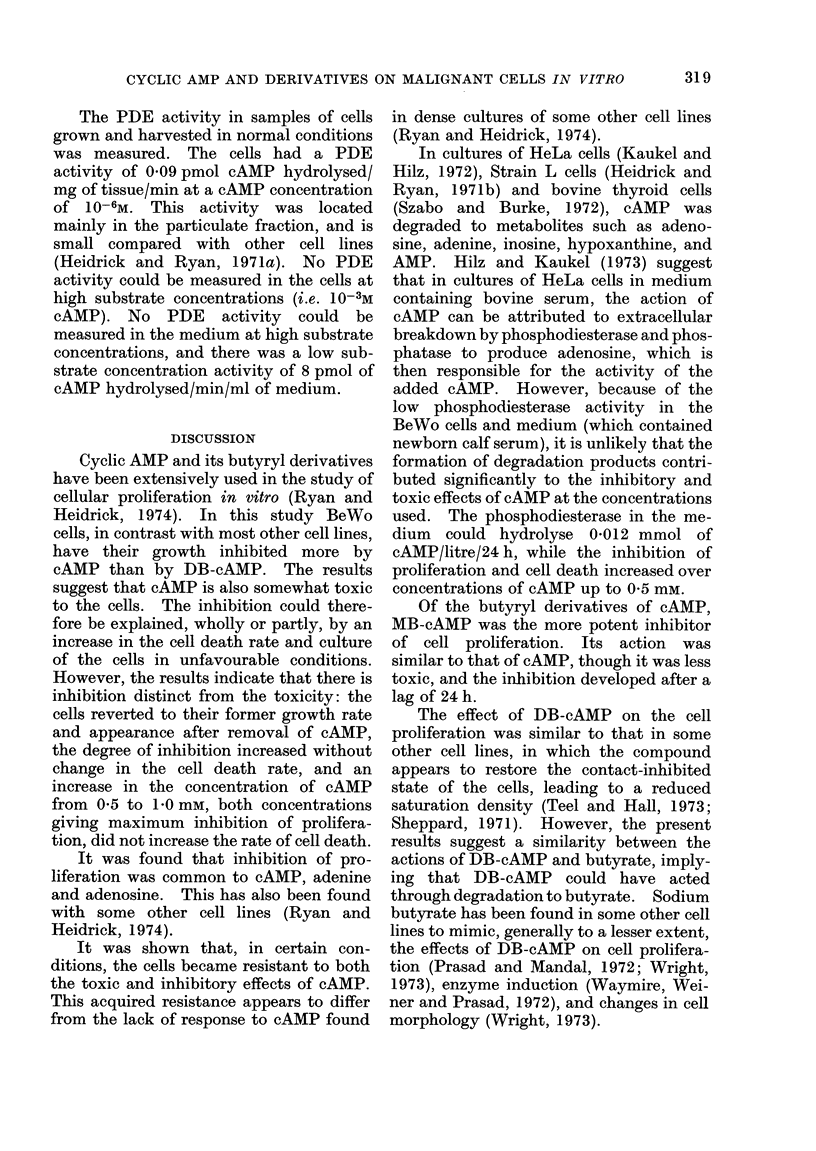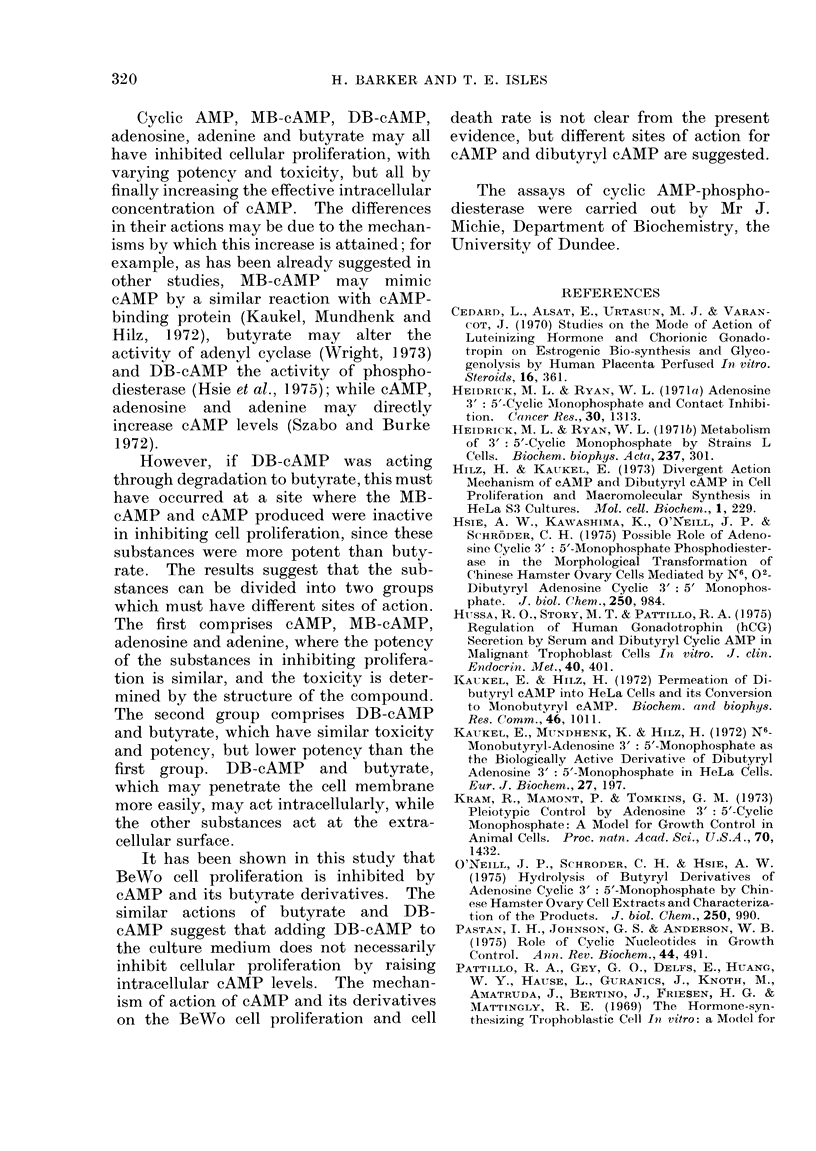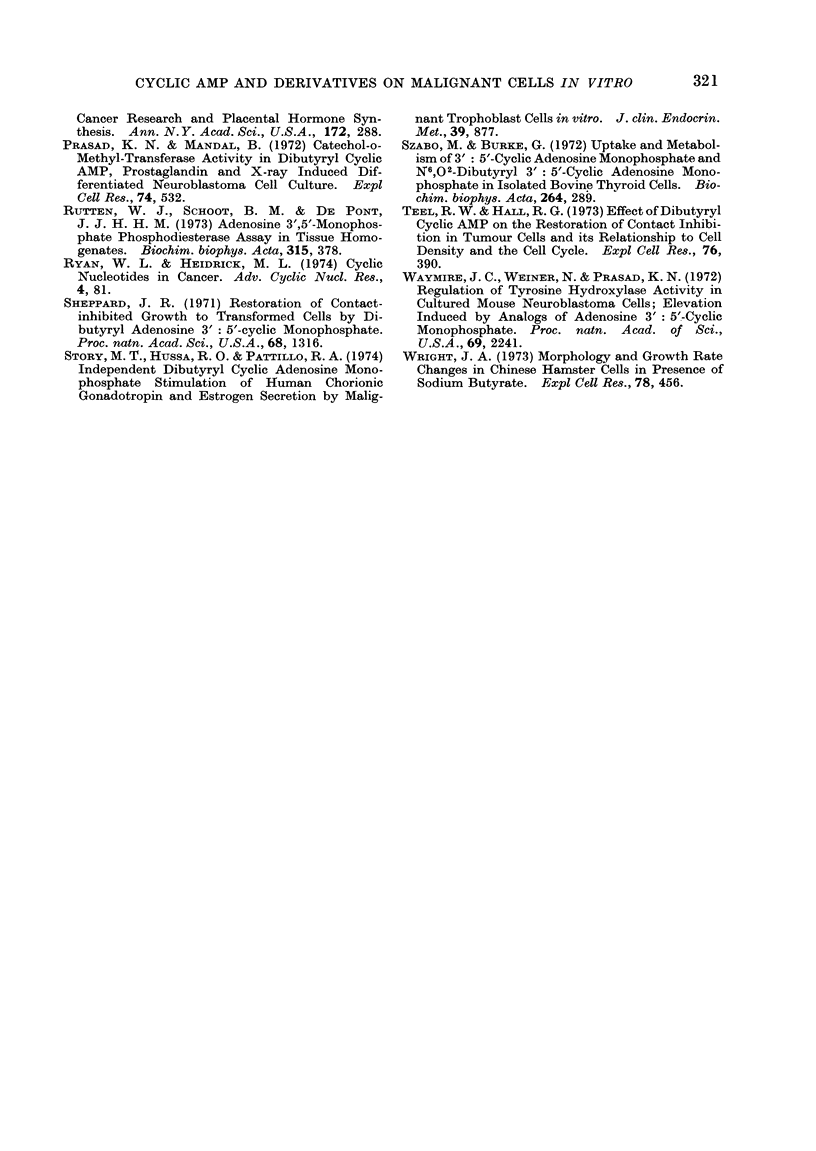# The actions of cyclic AMP, its butyryl derivatives and Na butyrate on the proliferation of malignant trophoblast cells in vitro.

**DOI:** 10.1038/bjc.1977.45

**Published:** 1977-03

**Authors:** H. Barker, T. E. Isles

## Abstract

Cyclic AMP, and its derivatives N6-monobutyryl cyclic AMP and dibutyryl cyclic AMP, have been found to inhibit the proliferation of trophoblast cells of the BeWo cell line in vitro. Sodium butyrate (1 mM), a possible degradation product of the butyrate derivatives, also inhibited cell proliferation, giving similar growth rates to equimolar dibutyryl cyclic AMP. The inhibition by butyrate was however, not sufficient to account for the action of 1 mM N6-monobutyryl cycli AMP, which, like cyclic AMP, completely inhibited cell proliferation. The potency, specificity and toxicity of the substances were compared. The results suggest different modes of action for cyclic AMP and dibutyryl cyclic AMP.


*Br. J. Cancer* (1977) **35**, 314.

### THE ACTIONS OF CYCLIC AMP, ITS BUTYRYL DERIVATIVES AND Na BUTYRATE ON THE PROLIFERATION OF MALIGNANT TROPHOBLAST CELLS *IN VITRO*

H. BARKER AND T. E. ISLES

*From the Department of Biochemical Medicine, Ninewells Hospital, Dundee*

Received 13 August 1976 Accepted 27 October 1976

**Summary.—Cyclic AMP, and its derivatives $N^6$-monobutyryl cyclic AMP and dibutyryl cyclic AMP, have been found to inhibit the proliferation of trophoblast cells of the BeWo cell line *in vitro*. Sodium butyrate (1 mM), a possible degradation product of the butyrate derivatives, also inhibited cell proliferation, giving similar growth rates to equimolar dibutyryl cyclic AMP. The inhibition by butyrate was, however, not sufficient to account for the action of 1 mM $N^6$-monobutyryl cyclic AMP, which, like cyclic AMP, completely inhibited cell proliferation. The potency, specificity and toxicity of the substances were compared. The results suggest different modes of action for cyclic AMP and dibutyryl cyclic AMP.**

MANY cell lines have been found to be inhibited by 3′: 5′-cyclic adenosine monophosphate (cAMP) and its butyryl derivatives (Ryan and Heidrick, 1974; Pastan, Johnson and Anderson, 1975), and the intracellular levels of cAMP are thought to be involved in the control of cellular proliferation, growth and differentiation (Ryan and Heidrick, 1974; Kram, Mamont and Tomkins, 1973). Malignant and transformed cells in culture generally have lower intracellular levels of cAMP, and many laboratories have reported that dibutyryl cAMP (DB-cAMP) and agents which raise intracellular cAMP levels, can reverse many of the properties of transformed cells in culture to those of normal cells (Ryan and Heidrick, 1974). However, levels of cAMP in tumours *in vivo* are generally higher than or similar to those in corresponding normal cells (Ryan and Heidrick, 1974; Pastan *et al.*, 1975).

Trophoblast cells of the BeWo and the similar JAR cell lines, derived from malignant choriocarcinoma, have been used as models for cancer research and placental hormone production (Pattillo *et al.*, 1969). It has been suggested that the cAMP levels in trophoblast cells of the placenta may relate to their hormone production (Cedard *et al.*, 1970). It has been found that DB-cAMP stimulates the output of oestrogens and human chorionic gonadotrophin of trophoblast cells *in vitro* (Story, Hussa and Pattillo, 1974; Hussa, Story and Pattillo, 1975).

The aim of this paper is to report the action of cAMP on BeWo cell proliferation. In many previous studies on other cell lines, the dibutyryl derivative of cAMP has been a more active inhibitor of cell proliferation than cAMP itself, and reasons suggested have been resistance to hydrolysis by cAMP phosphodiesterase and easier entry into cells (Ryan and Heidrick, 1974; Hilz and Kaukel, 1973). However, in HeLa S3 cells (Hilz and Kaukel, 1973), bovine thyroid cells (Szabo and Burke, 1972), and Chinese hamster ovary cells (O'Neill, Schroder and Hsie, 1975), the $N^6$-monobutyryl derivative of cAMP, which accumulated intracellularly through deacylation of DB-cAMP, has been suggested as the main active component. Butyrate is also formed from this conversion. In this study, therefore, the actions of cAMP, its butyryl derivatives and sodium butyrate have been compared.

### MATERIALS AND METHODS

Trophoblast cells of the BeWo cell line were obtained from Professor Pattillo, the Marquette School of Medicine, Wisconsin, U.S.A. The cells were propagated at 37 °C on the surface of polystyrene Petri dishes, 13 mm deep and 50 mm diameter, with 3 air vents.

The medium, which was changed daily, was TC 199 (Wellcome Reagents Ltd, Beckenham), and was supplemented with sodium bicarbonate to a final concentration 1·5 g/l, and 100 ml/l of newborn calf serum (Flow Laboratories, Irvine). The atmosphere was 95% air, 5% $CO_2$.

The cells were removed for subculture or counting, by first washing with Ca- and Mg-free Earle's balanced salt solution (Flow Laboratories, Irvine) and then incubating with 2 ml of 0·25% trypsin (Wellcome Reagents Ltd, Beckenham) in the same solution for 20 min at 37 °C. Single-cell suspensions were obtained by agitation and counted in a Coulter counter.

The dead cells in suspension in the medium after a period of culture were counted by collection of the whole medium and washings of the cell layer.

Adenine, adenosine and their derivatives were obtained from the Sigma Chemical Company.

Cyclic AMP phosphodiesterase (PDE) was measured by the method of Rutten, Schoot and De Pont (1973). Incubation time was 70 min.

*BeWo cell morphology.*—The cells after subculture were immobile, and the proliferation of single cells gave rise to colonies which merged at high cell density. Under normal conditions, the cell population consisted of predominantly transparent, flattened and stellate cells, with many long cell processes linking the colonies.

Cells in suspension, after removal from the dish surface with trypsin, were transparent and viable in subculture. Cells in suspension over the cell layer during culture were opaque and were not viable when removed and cultured in new dishes. The dead cells generated during culture were therefore measured by counts of the cells in suspension in the medium.

*Precision of cell counting.*—In the experiments presented, the estimates of cell density under normal cultural conditions (controls) and in media containing additives are single determinations or means of duplicate or triplicate cultures.

An indication of the variation in determinations of cell density was obtained by counting the cell densities in normal cultures 6 days after subculture.

Mean cell density =
22·3 $\times$ $10^5$ cells per dish
Standard deviation =
0·90 $\times$ $10^5$ (number of cultures = 25)
Coefficient of variation = 4·0%

The variation in counts of dead cells was estimated from the counts of dead cells in duplicate cultures throughout the study.

The differences between duplicates were random over the range of cell counts.

Mean difference between duplicates =
0·095 $\times$ $10^5$ cells per dish
Standard deviation = 0·105 $\times$ $10^5$
(number of duplicate cultures = 55)

### RESULTS

Medium containing cAMP inhibited the proliferation of BeWo cells. The response was dose-related (Table I). In 1·0 and 0·5 mM cAMP, the cell number increased by 9% and 19% respectively, compared to the 280% increase in the density of control cultures over a 3-day incubation.

All concentrations of cAMP used gave rise to changes in the appearance of the cells. There was progressively more rounding with fewer cell processes, as cAMP concentration was increased. At concentrations of 0·25 mM cAMP and above, the cells appeared more opaque than controls and contained more vacuoles. The numbers of dead cells were increased in all concentrations of cAMP (Table I), but reached a maximum at a concentration of 0·13 mM cAMP and above.

Removal of the cAMP after 2 days, and subsequent culture in normal medium, led to a reversion to the growth rate of control cultures (Fig. 1). The cells also reverted to their normal morphology.

When the cells were cultured for longer periods in concentrations of cAMP producing maximum inhibition of proliferation, after 4 to 6 days there was a

TABLE I.—*The Effect of Culture in Medium Containing cAMP on Cell Density and Cell Death*

| cAMP conc. mM | Cells ($\times 10^5$/dish) | Dead cells ($\times 10^5$/dish) | % Dead cells |
|---|---|---|---|
| 1·0 | 5·2 | 0·89 | 15 |
| 0·5 | 5·6 | 1·2 | 18 |
| 0·25 | 7·9 | 1·0 | 11 |
| 0·13 | 10 | 1·2 | 11 |
| 0·063 | 12 | 0·60 | 4·8 |
| 0·032 | 16 | 0·53 | 3·2 |
| 0 | 18 | 0·25 | 1·4 |

Cells at a density of $4{\cdot}7 \times 10^5$ cells/dish (3 days after subculture) were transferred to medium containing cAMP in concentrations from 0 to 1·0 mM. The medium was changed daily, and the cells were harvested after 3 days. The dead cells which had accumulated in the media over the preceding 24 h were counted.

FIG. 1.—Effect of incubation in medium containing cAMP, and its subsequent removal, on cell density. Medium containing cAMP (0·5 mM) was added to cells at a density of $2{\cdot}2 \times 10^5$/dish, 3 days after subculture. In some dishes, the cAMP was removed and replaced by normal medium and in others, incubation of the cultures in cAMP continued. Controls were maintained in normal medium throughout. Medium changed daily.

FIG. 2.—Effect of incubation in medium containing cAMP on the growth curves of initially dense cultures. Three days after subculture, cells of density $7{\cdot}8 \times 10^5$/dish were subsequently maintained in medium containing 0·5 △, 0·2 ▲, 0·1 ○ and 0 (controls) ● mM cAMP, with daily medium changes.

decrease in the cell number as the remaining cells were killed (*e.g.* Fig. 4).

The action of cAMP on denser cultures ($7{\cdot}8 \times 10^5$ cells per dish) was studied (Fig. 2). In 0·1, 0·2 and 0·5 mM cAMP there was a period of inhibition of cell proliferation followed by apparent recovery, with a growth rate similar to that of the controls. There was also a decrease in the numbers of dead cells in all concentrations of cAMP, after 3 days. The appearance of the cells changed during the culture period. After 2 days, cells in 0·5 mM cAMP grew in small colonies of smooth, rounded, opaque cells, with much of the space between the colonies empty. Cultures in the lower concentrations of cAMP were similar, but the colonies were larger. However, after 4 days' culture in cAMP the cells were no longer smooth and rounded, but clear cell processes were spreading into the empty spaces around the colonies. It appeared therefore that, in these conditions, the cells acquired resis-

FIG. 3.—Effect of incubation in medium containing adenine, adenosine, AMP, ATP or cAMP, on the cell density. Three days after subculture, cells of density 3·0 × $10^5$/dish were subsequently incubated in medium containing 1 mM of adenine, adenosine, AMP, ATP or cAMP or normal medium (controls) for 4 days, with daily medium changes.

tance to the toxic and inhibitory action of cAMP which occurred during the earlier period of culture.

The actions of adenine, adenosine, adenosine 3′: 5′-monophosphate (AMP) and adenosine 3′: 5′-triphosphate (ATP) were compared with that of cAMP (Fig. 3). Incubation in AMP and ATP led to a rapid decline in the numbers of viable cells, while adenine and adenosine had a similar action to cAMP, by inhibiting proliferation of the cells, with little change in cell density over the period of culture.

Table II shows the numbers of dead cells produced by incubation in the test substances, expressed as a percentage of the total cell counts, providing an indication of toxicity. All the test substances led to an increase in the rate of cell death. Table III compares the rates of proliferation of control cultures and cultures incubated with the test substances, including the numbers of dead cells in the total cell count. AMP and ATP, which are most toxic (Table II) also inhibited proliferation most (Table III). However, there is no clear relationship between inhibition and the toxicity of cAMP, adenine and adenosine; for example, the rate of cell proliferation in cAMP decreased from 96% after the first 48-h period to 20% after the second, but with no corresponding increase in cell death.

The actions of the related compounds dibutyryl cAMP (DB-cAMP) and $N^6$-monobutyryl cAMP (MB-cAMP) were investigated (Fig. 4 and Table IV). MB-cAMP was the more potent, and after 24 h there was almost complete inhibition of cell proliferation. The number of dead cells in 1 mM MB-cAMP was greater than in control cultures, but less than in 1 mM cAMP at about the same cell density (Table IV). The cells were cul-

TABLE II.—*Effect of Culture in Medium Containing Adenine, Adenosine, AMP, ATP, or cAMP on Cell Death*

| Additives (1 mM) | Dead cells × $10^5$/dish | | | | Dead cells as % of total | |
|---|---|---|---|---|---|---|
| | Day 1 | Day 2 | Day 3 | Day 4 | Day 2 | Day 4 |
| None (control) | 0·53 | 0·37 | 0·44 | 0·36 | 10 | 4·0 |
| cAMP | 1·2 | 0·99 | 1·1 | 0·79 | 40 | 46 |
| Adenine | 0·23 | 0·63 | 1·4 | 1·2 | 17 | 40 |
| Adenosine | 1·2 | 1·2 | 1·5 | 0·92 | 44 | 42 |
| AMP | 1·5 | 1·6 | 0·80 | 0·63 | 64 | 59 |
| ATP | 0·76 | 1·0 | 0·55 | 0·80 | 47 | 69 |

The cells were cultured under the conditions outlined in Fig. 3. After the addition of the test substances, the dead cells which accumulated in the media were counted. The cell densities after 2 and 4 days' incubation were measured. The dead cells generated over each 48 h period are expressed as percentages of the total cells.

tured for 6 days in 1 mM MB-cAMP without change in appearance, or decrease in the density, of the cells.

The rates of proliferation in 1 mM DB-cAMP or sodium butyrate were similar, after an initial delay in the action of DB-cAMP, leading to proliferation rates between the controls and 1 mM MB-cAMP. The cell death in DB-cAMP was similar to that in MB-cAMP, and there were also no morphological changes in the cells. A plateau of cell density of about $25 \times 10^5$

FIG. 4.—Effect of incubation in medium containing cAMP, MB-cAMP, DB-cAMP, or Na butyrate on cell density. Three days after subculture, cells of density $3 \cdot 3 \times 10^5$/dish were subsequently maintained in medium containing 1 mM of MB-cAMP, DB-cAMP or Na butyrate, or normal medium (controls) for 6 days, with daily medium changes. Medium containing 1 mM of cAMP was added to some control cultures 4 days after subculture, and they were then maintained in cAMP for 6 days, with daily medium changes.

TABLE III.—*Effect of Culture in Medium Containing Adenine, Adenosine, AMP, ATP, or cAMP on the Rates of Cell Proliferation*

| Additives (1 mM) | % increase in cell number between Days 0 and 2 | % increase in cell number between Days 2 and 4 |
|---|---|---|
| None (control) | 210 | 151 |
| cAMP | 96 | 20 |
| Adenine | 79 | 47 |
| Adenosine | 89 | 95 |
| AMP | 69 | 38 |
| ATP | 33 | 0 |

The cells were cultured under the conditions outlined in Fig. 3. The cell number is the sum of the cell density and the total number of dead cells generated over the 48-h period.

cells per dish was reached in DB-cAMP. In another experiment, with an initial cell density of $2 \cdot 2 \times 10^5$ cells per dish, the slopes of the growth curves of cells grown in 1 mM DB-cAMP or Na butyrate were the same between 1 and 6 days after addition, until both reached the same plateau of $25 \times 10^5$ cells per dish. Equimolar butyric acid had the same action as Na butyrate.

Increasing the concentration of glucose in the medium from 5·4 to 17·6 mM did not alter the growth rates in normal, DB-cAMP supplemented, and cAMP-supplemented medium.

TABLE IV.—*Effect of Culture in Medium Containing MB-cAMP, DB-cAMP, Na Butyrate or cAMP on Cell Death*

| | Dead cells $\times 10^5$/dish | |
|---|---|---|
| Additives (1 mM) | 2 days' incubation | 4 days' incubation |
| None (control) | 0·23 | 0·40 |
| MB-cAMP | 0·49 | 0·78 |
| DB-cAMP | 0·34 | 0·73 |
| Na-butyrate | 0·71 | 1·3 |
| cAMP (added day 1) | 1·5 | 2·0 |

The cells were cultured under the conditions outlined in Fig. 4. Dead cells which had accumulated in the medium during the preceding 24 h were counted.

The PDE activity in samples of cells grown and harvested in normal conditions was measured. The cells had a PDE activity of 0·09 pmol cAMP hydrolysed/mg of tissue/min at a cAMP concentration of $10^{-6}$M. This activity was located mainly in the particulate fraction, and is small compared with other cell lines (Heidrick and Ryan, 1971*a*). No PDE activity could be measured in the cells at high substrate concentrations (*i.e.* $10^{-3}$M cAMP). No PDE activity could be measured in the medium at high substrate concentrations, and there was a low substrate concentration activity of 8 pmol of cAMP hydrolysed/min/ml of medium.

### DISCUSSION

Cyclic AMP and its butyryl derivatives have been extensively used in the study of cellular proliferation *in vitro* (Ryan and Heidrick, 1974). In this study BeWo cells, in contrast with most other cell lines, have their growth inhibited more by cAMP than by DB-cAMP. The results suggest that cAMP is also somewhat toxic to the cells. The inhibition could therefore be explained, wholly or partly, by an increase in the cell death rate and culture of the cells in unfavourable conditions. However, the results indicate that there is inhibition distinct from the toxicity: the cells reverted to their former growth rate and appearance after removal of cAMP, the degree of inhibition increased without change in the cell death rate, and an increase in the concentration of cAMP from 0·5 to 1·0 mM, both concentrations giving maximum inhibition of proliferation, did not increase the rate of cell death.

It was found that inhibition of proliferation was common to cAMP, adenine and adenosine. This has also been found with some other cell lines (Ryan and Heidrick, 1974).

It was shown that, in certain conditions, the cells became resistant to both the toxic and inhibitory effects of cAMP. This acquired resistance appears to differ from the lack of response to cAMP found in dense cultures of some other cell lines (Ryan and Heidrick, 1974).

In cultures of HeLa cells (Kaukel and Hilz, 1972), Strain L cells (Heidrick and Ryan, 1971b) and bovine thyroid cells (Szabo and Burke, 1972), cAMP was degraded to metabolites such as adenosine, adenine, inosine, hypoxanthine, and AMP. Hilz and Kaukel (1973) suggest that in cultures of HeLa cells in medium containing bovine serum, the action of cAMP can be attributed to extracellular breakdown by phosphodiesterase and phosphatase to produce adenosine, which is then responsible for the activity of the added cAMP. However, because of the low phosphodiesterase activity in the BeWo cells and medium (which contained newborn calf serum), it is unlikely that the formation of degradation products contributed significantly to the inhibitory and toxic effects of cAMP at the concentrations used. The phosphodiesterase in the medium could hydrolyse 0·012 mmol of cAMP/litre/24 h, while the inhibition of proliferation and cell death increased over concentrations of cAMP up to 0·5 mM.

Of the butyryl derivatives of cAMP, MB-cAMP was the more potent inhibitor of cell proliferation. Its action was similar to that of cAMP, though it was less toxic, and the inhibition developed after a lag of 24 h.

The effect of DB-cAMP on the cell proliferation was similar to that in some other cell lines, in which the compound appears to restore the contact-inhibited state of the cells, leading to a reduced saturation density (Teel and Hall, 1973; Sheppard, 1971). However, the present results suggest a similarity between the actions of DB-cAMP and butyrate, implying that DB-cAMP could have acted through degradation to butyrate. Sodium butyrate has been found in some other cell lines to mimic, generally to a lesser extent, the effects of DB-cAMP on cell proliferation (Prasad and Mandal, 1972; Wright, 1973), enzyme induction (Waymire, Weiner and Prasad, 1972), and changes in cell morphology (Wright, 1973).

Cyclic AMP, MB-cAMP, DB-cAMP, adenosine, adenine and butyrate may all have inhibited cellular proliferation, with varying potency and toxicity, but all by finally increasing the effective intracellular concentration of cAMP. The differences in their actions may be due to the mechanisms by which this increase is attained; for example, as has been already suggested in other studies, MB-cAMP may mimic cAMP by a similar reaction with cAMP-binding protein (Kaukel, Mundhenk and Hilz, 1972), butyrate may alter the activity of adenyl cyclase (Wright, 1973) and DB-cAMP the activity of phosphodiesterase (Hsie *et al.*, 1975); while cAMP, adenosine and adenine may directly increase cAMP levels (Szabo and Burke 1972).

However, if DB-cAMP was acting through degradation to butyrate, this must have occurred at a site where the MB-cAMP and cAMP produced were inactive in inhibiting cell proliferation, since these substances were more potent than butyrate. The results suggest that the substances can be divided into two groups which must have different sites of action. The first comprises cAMP, MB-cAMP, adenosine and adenine, where the potency of the substances in inhibiting proliferation is similar, and the toxicity is determined by the structure of the compound. The second group comprises DB-cAMP and butyrate, which have similar toxicity and potency, but lower potency than the first group. DB-cAMP and butyrate, which may penetrate the cell membrane more easily, may act intracellularly, while the other substances act at the extracellular surface.

It has been shown in this study that BeWo cell proliferation is inhibited by cAMP and its butyrate derivatives. The similar actions of butyrate and DB-cAMP suggest that adding DB-cAMP to the culture medium does not necessarily inhibit cellular proliferation by raising intracellular cAMP levels. The mechanism of action of cAMP and its derivatives on the BeWo cell proliferation and cell death rate is not clear from the present evidence, but different sites of action for cAMP and dibutyryl cAMP are suggested.

The assays of cyclic AMP-phosphodiesterase were carried out by Mr J. Michie, Department of Biochemistry, the University of Dundee.

### REFERENCES

CEDARD, L., ALSAT, E., URTASUN, M. J. & VARANCOT, J. (1970) Studies on the Mode of Action of Luteinizing Hormone and Chorionic Gonadotropin on Estrogenic Bio-synthesis and Glycogenolysis by Human Placenta Perfused *In vitro*. *Steroids*, **16**, 361.

HEIDRICK, M. L. & RYAN, W. L. (1971*a*) Adenosine 3′ : 5′-Cyclic Monophosphate and Contact Inhibition. *Cancer Res.*, **30**, 1313.

HEIDRICK, M. L. & RYAN, W. L. (1971*b*) Metabolism of 3′ : 5′-Cyclic Monophosphate by Strains L Cells. *Biochem. biophys. Acta*, **237**, 301.

HILZ, H. & KAUKEL, E. (1973) Divergent Action Mechanism of cAMP and Dibutyryl cAMP in Cell Proliferation and Macromolecular Synthesis in HeLa S3 Cultures. *Mol. cell. Biochem.*, **1**, 229.

HSIE, A. W., KAWASHIMA, K., O'NEILL, J. P. & SCHRÖDER, C. H. (1975) Possible Role of Adenosine Cyclic 3′ : 5′-Monophosphate Phosphodiesterase in the Morphological Transformation of Chinese Hamster Ovary Cells Mediated by $N^6$, $O^2$-Dibutyryl Adenosine Cyclic 3′ : 5′ Monophosphate. *J. biol. Chem.*, **250**, 984.

HUSSA, R. O., STORY, M. T. & PATTILLO, R. A. (1975) Regulation of Human Gonadotrophin (hCG) Secretion by Serum and Dibutyryl Cyclic AMP in Malignant Trophoblast Cells *In vitro*. *J. clin. Endocrin. Met.*, **40**, 401.

KAUKEL, E. & HILZ, H. (1972) Permeation of Dibutyryl cAMP into HeLa Cells and its Conversion to Monobutyryl cAMP. *Biochem. and biophys. Res. Comm.*, **46**, 1011.

KAUKEL, E., MUNDHENK, K. & HILZ, H. (1972) $N^6$-Monobutyryl-Adenosine 3′ : 5′-Monophosphate as the Biologically Active Derivative of Dibutyryl Adenosine 3′ : 5′-Monophosphate in HeLa Cells. *Eur. J. Biochem.*, **27**, 197.

KRAM, R., MAMONT, P. & TOMKINS, G. M. (1973) Pleiotypic Control by Adenosine 3′ : 5′-Cyclic Monophosphate: A Model for Growth Control in Animal Cells. *Proc. natn. Acad. Sci., U.S.A.*, **70**, 1432.

O'NEILL, J. P., SCHRODER, C. H. & HSIE, A. W. (1975) Hydrolysis of Butyryl Derivatives of Adenosine Cyclic 3′ : 5′-Monophosphate by Chinese Hamster Ovary Cell Extracts and Characterization of the Products. *J. biol. Chem.*, **250**, 990.

PASTAN, I. H., JOHNSON, G. S. & ANDERSON, W. B. (1975) Role of Cyclic Nucleotides in Growth Control. *Ann. Rev. Biochem.*, **44**, 491.

PATTILLO, R. A., GEY, G. O., DELFS, E., HUANG, W. Y., HAUSE, L., GURANICS, J., KNOTH, M., AMATRUDA, J., BERTINO, J., FRIESEN, H. G. & MATTINGLY, R. E. (1969) The Hormone-synthesizing Trophoblastic Cell *In vitro*: a Model for

Cancer Research and Placental Hormone Synthesis. *Ann. N.Y. Acad. Sci., U.S.A.*, **172**, 288.

PRASAD, K. N. & MANDAL, B. (1972) Catechol-o-Methyl-Transferase Activity in Dibutyryl Cyclic AMP, Prostaglandin and X-ray Induced Differentiated Neuroblastoma Cell Culture. *Expl Cell Res.*, **74**, 532.

RUTTEN, W. J., SCHOOT, B. M. & DE PONT, J. J. H. H. M. (1973) Adenosine 3′,5′-Monophosphate Phosphodiesterase Assay in Tissue Homogenates. *Biochim. biophys. Acta*, **315**, 378.

RYAN, W. L. & HEIDRICK, M. L. (1974) Cyclic Nucleotides in Cancer. *Adv. Cyclic Nucl. Res.*, **4**, 81.

SHEPPARD, J. R. (1971) Restoration of Contact-inhibited Growth to Transformed Cells by Dibutyryl Adenosine 3′ : 5′-cyclic Monophosphate. *Proc. natn. Acad. Sci., U.S.A.*, **68**, 1316.

STORY, M. T., HUSSA, R. O. & PATTILLO, R. A. (1974) Independent Dibutyryl Cyclic Adenosine Monophosphate Stimulation of Human Chorionic Gonadotropin and Estrogen Secretion by Malignant Trophoblast Cells *in vitro*. *J. clin. Endocrin. Met.*, **39**, 877.

SZABO, M. & BURKE, G. (1972) Uptake and Metabolism of 3′ : 5′-Cyclic Adenosine Monophosphate and $N^6,O^2$-Dibutyryl 3′ : 5′-Cyclic Adenosine Monophosphate in Isolated Bovine Thyroid Cells. *Biochim. biophys. Acta*, **264**, 289.

TEEL, R. W. & HALL, R. G. (1973) Effect of Dibutyryl Cyclic AMP on the Restoration of Contact Inhibition in Tumour Cells and its Relationship to Cell Density and the Cell Cycle. *Expl Cell Res.*, **76**, 390.

WAYMIRE, J. C., WEINER, N. & PRASAD, K. N. (1972) Regulation of Tyrosine Hydroxylase Activity in Cultured Mouse Neuroblastoma Cells; Elevation Induced by Analogs of Adenosine 3′ : 5′-Cyclic Monophosphate. *Proc. natn. Acad. of Sci., U.S.A.*, **69**, 2241.

WRIGHT, J. A. (1973) Morphology and Growth Rate Changes in Chinese Hamster Cells in Presence of Sodium Butyrate. *Expl Cell Res.*, **78**, 456.